# Preliminary study on the effects of carbon dioxide and nitrogen pneumoperitoneums on endometriotic lesions

**DOI:** 10.1007/s00404-011-2206-1

**Published:** 2012-03-23

**Authors:** Lingyun Hu, Li-an Li, Yali Li

**Affiliations:** Department of Obstetrics and Gynecology, PLA General Hospital, Beijing, 100853 China

**Keywords:** Endometriosis, Pneumoperitoneum, Carbon dioxide, Nitrogen

## Abstract

**Objective:**

To determine the effects of carbon dioxide (CO_2_) and nitrogen (N_2_) pneumoperitoneums on endometriosis (EMs) lesions.

**Methods:**

Female Wistar rats were randomized into the following 3 groups: CO_2_ (*N* = 20), N_2_ (*N* = 22) and air pneumoperitoneums (*N* = 9). After 5 weeks of establishment models, do the pneumoperitoneums. Then measure the size of EMs lesions and the related factors of serum and tissue after 1, 2, and 4 weeks of pneumoperitoneums.

**Results:**

(1) One week after the pneumoperitoneum was established, the EMs lesions in the CO_2_ group were largest in volume, whereas at 4 weeks the EMs lesions in the CO_2_ group were smaller than the N_2_ group. (2) The level of ICAM-1 and TIMP-2 of serum in CO_2_ and N_2_ group after 2 weeks of pneumoperitoneum were higher than air group. (3) The expression of CD44v6, ICAM-1, MMP-2 and VEGF of tissue in CO_2_ and N_2_ group after 1, 2 and 4 weeks of pneumoperitoneum were lower than air group, TIMP-2 and ENS were higher than air group.

**Conclusion:**

After a CO_2_ pneumoperitoneum, EMs lesions were reduced in volume, suggesting an inhibitory effect on EMs lesions.

## Introduction

Endometriosis (EMs) is a common gynecologic condition, namely the presence of stromal and/or endometrial glandular epithelium implants in extra-uterine locations, possibly compromising several sites. It affects about 10% of childbearing age women and 3–5% of post-menopausal women. The most common site is ovary, the clinical symptoms are chronic pelvic pain, dysmenorrhea, menorrhagia, vibration pain, dyspareunia and dysuria, it may causes infertility. It is a benign disease, but the biological behavior mimics that of malignant tumor. Although there are drugs to treat in the future, the recurrence rate is still high (6.1–40%), with a rising trend [1, 2]. In recent years, the widespread application of laparoscopy in clinical practice not only provides accurate staging and diagnosis of EMs but also improves the chronic pelvic pain. CO_2_ is the insufflating medium of choice in contemporary laparoscopy due to the low expense, high solubility, flame retardant properties, and low possibility of air embolism. However, the previous studies on human colon and hepatic cancer cell lines treated with CO_2_ revealed that the gas can promote cell growth, leading to the spread and implantation of cancer cells [3–5]. A study in China revealed a decreased trend in the EMs recurrence following laparoscopy [6], another study involving HO8910 and SKOV3 ovarian cancer cell lines showed that cell growth is slowed with the increase in pressure and duration of the CO_2_ pneumoperitoneum [7]. Therefore, the effect of CO_2_ on the spread of tumor is still under debate. There are no reports regarding the biological effects of pneumoperitoneums on the EMs lesions and recurrence following laparoscopic treatment. This study aimed to explore the impact of different insufflating gases on EMs lesions.

## Materials and methods

### Materials

#### Experimental animals

Fifty-six female Wistar rats weighing 190–210 g were purchased from the Laboratory Animal Center of the Academy of Military Medical Sciences.

#### Reagents and equipment

The following reagents and equipment pieces were used: MK3 microplate reader (Labsystems, Finland); ELISA kit (R&D Systems, USA); and XW-80A vortex shaker, F6/10 homogenizer, 1-15 K and 3K15 refrigerated microcentrifuge, ABI 7500 real-time PCR instrument, and SYBR Green PCR kit.

### Methods

#### Establishment of an animal model for EMs

One day prior to the surgery, all animals were administered estradiol benzoate (0.1 ml/kg intramuscular) to synchronize the estrous cycle. After anesthesia, one side of the uterus was removed. The removed portion of the uterus was irrigated with normal saline at 37°C and cut sagittally to expose the uterine cavity. After the removal of the uterine serosa and myometrium, the endometrium was isolated, separated into two 5 mm × 10 mm parts, and fixed onto the superior-medial abdominal sides of the bilateral pelvic wall. Metronidazole and penicillin were given as anti-infection treatments.

#### Grouping and observation parameters

The rats were randomized into three groups according to the type of intervention (CO_2_, N_2_, and air). Five weeks after establishment of the animal model, pneumoperitoneums with CO_2_, N_2_, or air were administered (air pressure: 15 mmHg; time: 1 h). At weeks 1, 2, and 4 after the pneumoperitoneums were established, the sizes of the EMs lesions were measured, and serum and tissue molecular biomarkers (matrix metalloproteinase-2 MMP-2, tissue inhibitor of metalloproteinase-1 TIMP-1, CD44, ICAM-1, endostatin/vascular endothelial growth factor ENS/VEGF, and tumor necrosis factor-alpha TNF-α) were tested.

#### Test methods

Six-to-eight ml of intraventricular blood was aspired via intracardiac puncture. After centrifugation at 3,500 rpm/min, the upper layer of serum was collected, refrigerated at −80°C, and analyzed using ELISA. The EMs lesions were removed and irrigated with normal saline. One part of the lesion was frozen in liquid nitrogen for RT-PCR analysis, and the other part was fixed in formalin solution for pathologic examination to confirm the existence of EMs tissue.

#### RT-PCR primer design

The following primers were used:

- ENS: sense: ATCGTCAACCTGAAGGATG, antisense: GTCTGAGCCATGCCATAC.
- CD44v6: sense: CCTAATAGCACAACAGAAGAAG, antisense: GATCCATGAGTCACAGTGTC
- ICAM-1: sense: TGTCGGTGCTCAGGTATC, antisense: AGTGGTCTGCTGTCTTCC
- MMP-2: sense: GATATAGCCTATTCCTTGTG, antisense: TGTCAGTATCAGCATCAG
- TIMP-2: sense: TTACCCTCTGTGACTTTATTG, antisense: CCATTGATGCTCTTCTCTG
- VEGF: sense: TGGACCCTGGCTTTACTG, antisense: GGACGGCTTGAAGATATACTC
- β-Actin: sense: TATCGGCAATGAGCGGTTC, antisense: AGCACTGTGTTGGCATAGAG

### Statistical analysis

Software (Excel’97) was used to collect data, the data expressed as mean ± SD, and the analysis of variance for comparison of categorical variables. Significance was assumed when *P* was less than 0.05.

## Results

### EMs rat model was established successfully

The CO_2_ group (*N* = 20) had a success rate of 100% (20/20), the N_2_ group (*N* = 22) had a success rate of 72.7% (16/22), and the air group (*N* = 9) had a success rate of 88.9% (8/9). The pathologic examinations confirmed the existence of endometrial tissues and glands.

### The change of EMs lesions sizes

One week after pneumoperitoneum, the CO_2_ group demonstrated the largest EMs lesion based on volume (87.763 ± 10.138 mm^3^) compared to the N_2_ and air groups (*p* < 0.05). Four weeks after the pneumoperitoneum, the lesions in the CO_2_ group were smaller than those in the N_2_ group. In the CO_2_ group, the lesions obtained after 1 week were larger than it being in the second and fourth week.

**Fig. 1:**
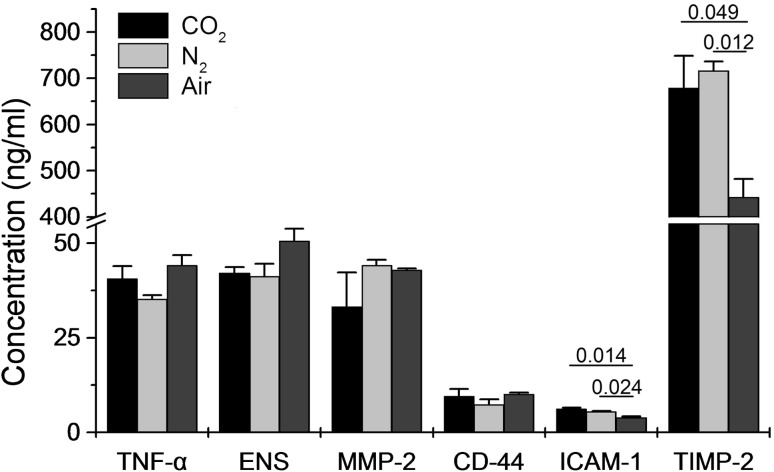
The level of adhesion-related factors (CD44, ICAM-1), invasion-related factor (MMP-2, TIMP-2) and angiogenesis-related factors (VEGF and TNF-α) in serum is changed after different pneumoperitoneum. Data are presented as mean ± SD

The serum ICAM-1 and TIMP-2 increase to show that capability of adhesion, invasion and angiogenesis after different pneumoperitoneum, we tested whether the level of CD44, ICAM-1, MMP-2, TIMP-2, VEGF and TNF-α change in serum. The ICAM-1 is adhesion-related factor, 6.22 ± 0.24 ng/L in CO_2_ group, 5.49 ± 0.19 ng/L in N_2_ group and 3.93 ± 0.31 ng/L in air group of the second week. The TIMP-2 is anti-invasion factor, 679.00 ± 69.74 ng/L in CO_2_ group, 716.91 ± 19.71 ng/L in N_2_ group and 442.49 ± 39.42 ng/L in air group of the second week. The CO_2_ and N_2_ groups had higher levels of ICAM-1 and TIMP-2 in serum than the air group (ICAM-1: *P* = 0.014, *P* = 0.024; TIMP-2: *P* = 0.049, *P* = 0.012; Fig. 1). The other markers revealed no statistically significant between group. The results indicated that the capability of adhesion and anti-invasion was improved after 2 weeks of CO_2_ and N_2_ pneumoperitoneum, there was no difference between CO_2_ and N_2_ pneumoperitoneum. Thereby, we suggest pneumoperitoneum will affect the capability of adhesion and invasion, but the different media have no difference. However, with the change of time, six genes of CO_2_ and N_2_ pneumoperitoneum vary significantly.

### The changed level of CD44v6, ICAM-1, MMP-2, VEGF, TIMP-2, ENS in tissue of EMs lesion

**Fig. 2:**
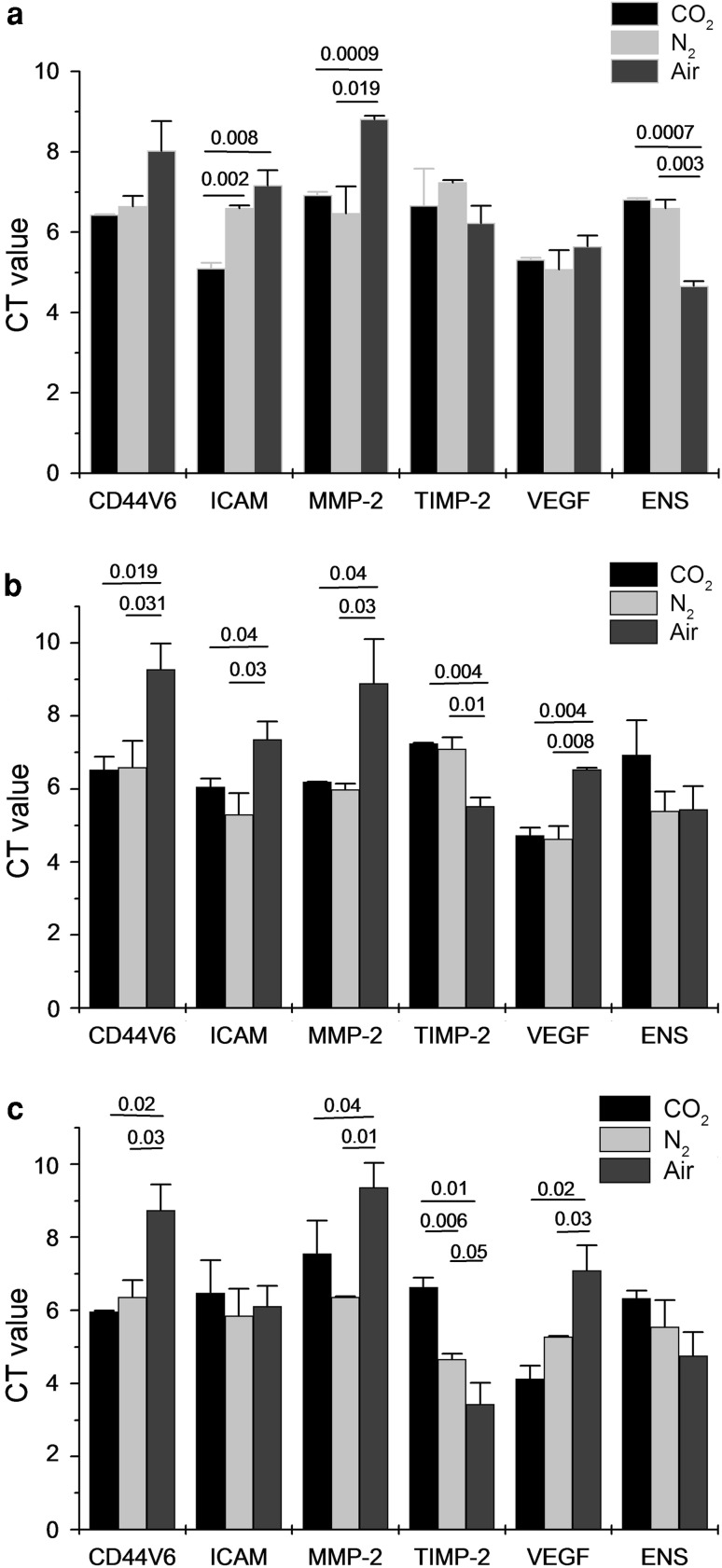
The adhesion-related factors (CD44v6, ICAM-1), invasion-related factor (MMP-2, TIMP-2) and angiogenesis-related factors (VEGF and ENS) in EMs lesion were changed after different pneumoperitoneum. Data are presented as mean ± SD

At weeks 1, 2, and 4, EMs tissues from the CO_2_ and N_2_ groups demonstrated significantly lower expressions of the adhesion-related factors (CD44v6 and ICAM-1), the invasion-related factor (MMP-2), and the vascular factor (VEGF), and significantly higher expressions of the anti-invasion factor (TIMP-2) and anti-angiogenesis factor (ENS), compared to the air group. In addition, the difference of ICAM-1, MMP-2, and ENS between CO_2_ and air group, N_2_ and air group were significant at 1 week (Fig. 2a); except ENS, the change of other six markers had significant difference at 2 weeks after pneumoperitoneum (Fig. 2b); the level of CD44v6, MMP-2, TIMP-2, and VEGF appeared obvious difference (Fig. 2c). Furthermore, the level of ICAM-1 was lower in CO_2_ group than N_2_ group at the first week, TIMP-2 was higher in N_2_ group than CO_2_ group. Therefore, we consider that pneumoperitoneum can reduced the capability of adhesion, invasion and angiogenesis in EMs lesion, and in some degree, the CO_2_ pneumoperitoneum was better than N_2_ pneumoperitoneum. In addition, the expression level of TIMP-2 decreased in N_2_ group and VEGF decreased in CO_2_ group at the fourth week after pneumoperitoneum. So, from the result, N_2_ decreased the capability of anti-invasion and CO_2_ decreased the capability of angiogenesis.

## Discussion

### Differences in various adhesion-related factors between different pneumoperitoneum interventions

Adhesion is the first step in which the endometrialis implanted in extra-uterine locations via menstrual blood. Cellular adhesion-related factors are molecules on the cellular surface that mediate cell–cell and cell-extracellular matrix adhesion. In the current study, we examined the level of ICAM-1 and CD44v6/CD44. Previous studies have shown that a high-pressure CO_2_ pneumoperitoneum can affect the synthesis of the hyaluronic acid complex, hence reducing adhesiveness [8]. CD44v6 is an isomer of CD44 and its relationship with epithelial tumors has become a research focus in recent years. A study on eutopic and ectopic endometrium suggested that both tissues express CD44 [9]. Compared to healthy control subjects, eutopic endometrium in EMs patients showed elevated expressions of CD44v6, CD44v7, CD44v8, and CD44v9 [10]. The presence of CD44 suggests that ectopic endometrium has the potential of peritoneal implantation. In the current study, there was no significant difference between-group in serum CD44, while the EMs tissues in the CO_2_ and N_2_ groups had higher serum ICAM-1 and lower CD44v6 and ICAM-1 expression compared to the air group. Our findings revealed that CO_2_ and N_2_ pneumoperitoneums inhibit EMs adhesion. The mechanism for this phenomenon may be related to lymphocyte homing, lymphocyte activation, and intracellular signal transduction. In addition, the expression of ICAM-1 in CO_2_ group was lower than in the N_2_ group after 1 week of pneumoperitoneums, which have significant statistic sense, and shows that CO_2_ as the medium of pneumoperitoneum is more effective to control the development and recurrence of endometriosis at early stage of post-operation.

### Differences in various invasion-related factors between different pneumoperitoneum interventions

Based on Sampson’s theory [11], invasion is the second step in EMs development. Overexpression of MMP-2 is related to invasion and metastasis of several tumors [12], while EMs also have invasive behavior. Based on the extant literature, patients with EMs have higher MMP-2 levels in the serum and peritoneal fluid compared to patients without EMs; the stroma and epithelium of ectopic endometrium also demonstrate increased MMP-2 expression [13]. In addition, genotype can affect risks for EMs. Specifically, women with the CC genotype of MMP-2-735 are at relatively increased risk for EMs [14]. Interestingly, women with the C/C homozygote genotype of TIMP-2-418 have significantly reduced MMP-2 expression [15]; TIMP-2 regulates MMP-2. Several studies had confirmed the correlation between MMP-2, TIMP-2, and several diseases [16]. In our study, after 1, 2, and 4 weeks of the pneumoperitoneum, EMs tissues in the CO_2_ and N_2_ groups had a lower expression of MMP-2 than the air group. Furthermore, the increase in TIMP-2 after a pneumoperitoneum provided another piece of evidence that CO_2_ and N_2_ pneumoperitoneums may limit invasiveness of ectopic endometrial tissues. The finding suggested that CO_2_ and N_2_ pneumoperitoneums can reduce invasiveness of EMs, thus inhibiting the post-operative recurrence and controlling further development of EMs. Second, at the fourth week after pneumoperitoneum, the TIMP-2 expression in CO_2_ group was significantly higher than N_2_ group, so we can speculate that CO_2_ as the medium may benefit to capability of anti-invasion in the long period of post-operation.

### Differences in the angiogenic factor between different pneumoperitoneum interventions

Angiogenesis plays a critical role in the metastasis VEGF is one of the most important agents affecting angiogenesis and can induce endothelial proliferation, capillary loop formation, and consequently new blood vessel formation can also increase capillary permeability. A previous study showed no difference in serum VEGF between patients with stage III-IV EMs and the control group [17]. TNF can facilitate EMs development by stimulating ectopic endometrial proliferation and local papillary growth. In the current study, EMs tissues in the CO_2_ and N_2_ groups had higher VEGF expression at the second and fourth week, significantly higher ENS expression at the first week. In the long run of the effect, the CO_2_ is better than N_2_, because the capability of angiogenesis by the expression of VEGF increased in the N_2_ group at the fourth week after pneumoperitoneum.

There was no difference in serum markers, except ICAM-1 and TIMP-2. Considering the fact that EMs lesions secrete these adhesion-, invasion-, and angiogenesis-related factors, which cannot be detected in the blood until having reached an accumulated threshold amount, RT-PCR is a more sensitive method by which to measure variations in such factors in EMs tissues.

From the results, we think that compared to air pneumoperitoneum, CO_2_ and N_2_ pneumoperitoneums inhibit EMs lesions not only with respect to lesion size but also with respect to other biomarkers, and the CO_2_ as medium of pneumoperitoneums is better than N_2_. Therefore, in addition to laparoscopic treatment for EMs, the choice of insufflating gas also has an impact on therapeutic outcome. In light of the effects on pain reduction and increased pregnancy rate [18], laparoscopic treatment for EMs is the preferred approach for non-parous women with desired fertility. During surgery, an intra-abdominal pressure of 14 mmHg should be maintained, while some increase in pressure may be required based on the status of the patient. Previous literature has shown that different intra-abdominal pressures exert varying degrees of influence on immune functions in experimental animals. Because a higher pneumoperitoneum pressure generates stronger inhibition on the immune response and further studies are needed to elucidate whether or not the same high intra-abdominal pressure may cause additional implantation and invasion of EMs cells.
